# Factors Associated With Newly Graduated Nurses' Work Engagement: Systematic Review of Quantitative Studies

**DOI:** 10.1111/jan.17069

**Published:** 2025-06-27

**Authors:** Hanna Ojala, Heli‐Maria Kuivila, Kristina Mikkonen, Erika Jarva, Jonna Juntunen

**Affiliations:** ^1^ Oulu University Hospital Oulu Finland; ^2^ Research Unit of Health Sciences and Technology, University of Oulu Oulu Finland; ^3^ Medical Research Center Oulu, Oulu University Hospital and University of Oulu Oulu Finland

**Keywords:** factors, newly graduated nurses, systematic review, work engagement

## Abstract

**Aim:**

To describe the factors associated with work engagement in newly graduated nurses.

**Design:**

Systematic review of original quantitative studies according to Joanna Briggs Institute guidelines.

**Methods:**

The systematic review utilised PEO inclusion criteria. Original peer‐reviewed quantitative studies were identified. Two researchers independently conducted a screening of study eligibility based on title, abstract, and full text. The JBI critical appraisal tool for analytical cross‐sectional studies was employed to perform a rigorous methodological quality assessment. The data was extracted, tabulated, and then analysed narratively.

**Data Sources:**

The literature search was conducted in November 2023 by screening four databases: Scopus, CINAHL (Ebsco), ProQuest, and Ovid Medline.

**Results:**

The review included 19 articles, presenting an overview of factors associated with newly graduated nurses' work engagement. Factors were classified into seven categories explaining supportive workplace, transition and orientation to workplace, competence and career development in nursing practice, personal and psychological characteristics, work environment characteristics, stress and challenges in a work environment, and satisfaction with work.

**Conclusions:**

To support newly graduated nurses' work engagement, nurse leaders should provide a supportive working environment and focus on new nurses' effective support systems in the workplace. Their abilities to develop and educate themselves need to be prioritised to enhance their knowledge and skills in nursing. Additionally, organisations should have policies and procedures to ensure quality orientation, and units need to implement transition and mentorship programmes.

**Implications for the Profession and/or Patient Care:**

This research could be valuable to health care when wanting to develop and improve work engagement, especially among newly graduated nurses. The economic significance of nurses' work engagement is evident, as the cost of nurse turnover is considerable. Reducing nurse turnover and improving retention relies on understanding the factors influencing nurses' decisions to leave the organisation and the profession.

**Impact:**

*What problem did the study address?* The global shortage of nurses, worsened by newly qualified nurses leaving the health sector, necessitates understanding factors influencing their work engagement; The factors associated with newly graduated nurses' work engagement were supportive work environment, transition and orientation to work, success and career development in nursing, personal and psychological characteristics, characteristics of the work environment, stress and challenges in the work environment, and job satisfaction. *Where and on whom will the research have an impact?* The results can be used by health care organisations to plan the preceptorship/mentoring programmes of new nurses. Identifying and understanding the factors associated with the retention of newly qualified nurses can help to attract and retain nurses and to promote the adaptation and integration of new nurses into healthcare organisations.

**Reporting Method:**

The YNEPR author checklist has been completed and implemented during this systematic review process. Also, the Prisma 2020 checklist has been used.

**Patient or Public Contribution:**

No patient or public contribution: systematic review.

**Trial Registration:**

PROSPERO number: CRD42023408705 (https://www.crd.york.ac.uk/PROSPERO/)

## 1 Introduction

Nurses' work engagement is a socially and professionally important issue. As the population ages, the need for care increases, and in the next 10 years, a quarter of nurses will leave the labour market due to retirement (Sohlman 2020). The shortage of nurses has become a global problem and is projected to increase in the future (WHO 2020). Work engagement is defined as “a positive, fulfilling, work‐related state of mind characterized by vigor, dedication, and absorption.” In this context, vigour refers to high energy levels, focused effort, and persistence in one's work; dedication refers to strong commitment and enthusiasm; and absorption refers to joyful involvement and the sensation of time passing quickly (Schaufeli et al. 2002, 74). In previous studies, work engagement is often measured using the Utrecht work engagement scale (UWES) (e.g., Adachi and Inaba 2022; Aydin et al. 2023). In healthcare, work engagement has been identified as a key factor promoting ethical nursing practice to ensure quality care and positive patient outcomes (Keyko et al. 2016).

Research shows that nurses remain dissatisfied with their working lives. Nurses value meaningful work. However, challenges to well‐being, working conditions, and the attractiveness of the profession prevent these expectations from being met. Compared to other healthcare professionals, the retention of nurses is a major global concern, and organisations face difficulties in recruiting and retaining nurses (Niskala et al. 2020). Nurses´ work engagement has been identified as related to job satisfaction (Yan et al. 2019), innovative work behaviours (Van Zyl et al. 2021), and work ability (Tomietto et al. 2019). Furthermore, the economic significance of nurses' work engagement is evident, as the cost of nurse turnover is considerable (Zhao et al. 2019). Reducing nurse turnover and improving retention relies on understanding the factors influencing nurses' decisions to leave the organisation and the profession (Kim and Yeo 2019).

Limited work experience, coupled with constant change and increasing complexity in the healthcare sector, often drives new nurses to consider changing jobs or leaving the profession. Different competence development initiatives, such as supportive orientation and preceptors' education, can foster newly graduated nurses' professional identity and commitment to healthcare (Lindfors et al. 2022b). Organisations should invest in the competence development of newly graduated nurses during their early careers, as skills such as utilising research knowledge, assessing patient care effectiveness, coordinating, organising, delegating, and prioritising evolve with experience. Ensuring that new nurses practise these competencies from the start is crucial for the future of nursing and the development of nursing care (Lindfors et al. 2022b).

Millennial nurses value positive professional relationships, teamwork, and encouraging leadership, as these factors contribute to their retention and professional growth (Waltz et al. 2020). Addressing the factors that lead nurses to leave the sector and improving retention rates enables the long‐term sustainability of the nursing workforce and the success of the organisation (Baumann et al. 2019). According to previous studies, newly graduated nurses leave their first job within 1.5–2.5 years of graduation, mostly for work‐related reasons. Recent systematic reviews have identified individual factors like physical and psychological health, professional identity, professional commitment, and development, and environmental factors like workplace culture, engagement, and management have been recognised as factors influencing newly graduated registered nurses' intention to leave the nursing profession (Brown et al. 2024).

The transition from student to nurse is a period when new nurses are in the process of exploring their place in the healthcare sector. Together with preparation provided by nursing education, the organisational factors in the healthcare workplace influence new graduate nurses' readiness for clinical work, the challenges they perceive, and their needs for learning and support (Sterner et al. 2023). Factors that support newly graduated nurses' transition are adequate communication between preceptors and new graduates, structured transition to practice and residency programs, preceptor competence and preparedness, emotional support from preceptors, and availability of mentorship in various forms. The main barriers to achieving optimum transition to practice are poor communication between preceptors and new graduates, inadequate time for one‐on‐one interaction with preceptors due to workload, and new graduates' perception of incompetence and inability to assimilate (Reebals et al. 2022). Well‐structured and supportive transition to practice programs increase newly graduated nurses' confidence and competence, which in turn increases job satisfaction (Weller‐Newton et al. 2022). There has been a relatively large amount of research on the work engagement of newly graduated nurses. Previous studies (i.e., Pericak et al. 2020; Kim and Kim 2022) have explored the reasons for turnover or work engagement, but the research data have not been systematically and consistently compiled into factors that inhibit and promote the work engagement of newly graduated nurses.

## 2 Aim

The aim of the systematic review was to describe the factors associated with work engagement in newly graduated nurses. The results of this study will provide healthcare organisations with information on what newly qualified nurses require to commit to their work so that they stay in nursing and how to increase the attractiveness of the nursing profession.

The research question was, “what factors are associated with newly graduated nurses' work engagement?”

## 3 Methods

### 3.1 Design

This systematic review was conducted following the guidelines of the JBI Manual for Evidence Synthesis—Systematic Review (Aromataris and Munn 2020). The research protocol was defined before the literature search.

### 3.2 Search Methods, Inclusion, and Exclusion Criteria

The research question and the keywords were defined using the PEO format (Munn et al. 2018). Inclusion criteria were defined according to the PEO format as follows: participants: newly graduated nurses from various primary and specialised health care settings; exposure of interest: factors affecting work engagement; and outcomes: engagement to nursing work and intentions to stay in the nursing profession and current nursing position. Exclusion criteria were also created according to the PEO format (Table 1). This systematic review searched for original, peer‐reviewed, quantitative studies that were cross‐sectional or cohort studies in terms of research methods and were published from 2013 to 2023. A time limit was set to this to provide the most recent research on the work engagement of newly graduated nurses. The global concern about the adequate number of graduated nurses has intensified. A report published in 2023 by the International Council of Nurses (ICN) highlights that the nursing shortage is a global health emergency. The World Health Organisation (WHO) has estimated that by 2030, nine million new nurses and midwives will be needed to meet the growing healthcare demands. Given the increasing urgency of this issue, we chose a 10‐year timeframe for this review to capture trends, policy responses, and challenges that have emerged over the past decade (Buchan and Catton 2023). Four databases were selected from which to retrieve original studies for the systematic review (Scopus, CINAHL(Ebsco), ProQuest, and Ovid Medline) (see Supporting Information S1). The database search was conducted at the end of November 2023.

**TABLE 1:** PEO inclusion and exclusion criteria.

PEO inclusion	PEO exclusion	Keywords
Participants = newly graduated nurses	Participants = other than newly graduated nurses, newly graduated midwives/public health nurses/other healthcare professionals	(“newly qualified” or “newly graduate” or newcomer* or “early career” or novice or “recent graduate”) or (MH “Novice Nurses”) AND nurs* or (MH “Practical Nurses”)
Exposure of interest = factors affecting work engagement	Exposure of interest = studies where factors affecting work engagement haven't been included	
Outcome = engagement in nursing work, intentions to stay in the nursing profession	Outcome = other than nursing work engagement	AND (MH “Work Engagement”) or (MH “Personnel Turnover”) or (MH “Personnel Retention”) or (“work engagement” or “intention to stay” or “intention to leave” or “turnover intention” or turnover)
Study design = Quantitative studies (e.g., analytical and descriptive cross‐sectional studies, cohort studies, correctional studies, correlational studies and mixed method studies) Languages: English, Finnish Timelimit: No Peer‐reviewed, original studies Peer‐reviewed original studies, cross‐sectional studies, cohort studies, correlational studies and mixed method studies	Study design = Qualitative studies, reviews, randomised controlled trials Languages: other than English or Finnish	AND NOT ((MH “Nonexperimental Studies+”) or “qualitative research” or “observational stud*” or “observational research”) AND NOT ((MH “Intervention Trials”) OR (MH “Experimental Studies+”) OR (MH “Randomised Controlled Trials+”) OR (interven* or “randomi?ed. controlled trial*” or experimental or “trial stud*”)) OR ((MH “Qualitative Studies+”) OR (MH “Systematic Review”) OR (MH “Meta Analysis”) or (“qualitative stud*” or “qualitative research” or “review” or “meta‐analysis”))

### 3.3 Search Outcome

**FIGURE 1:**
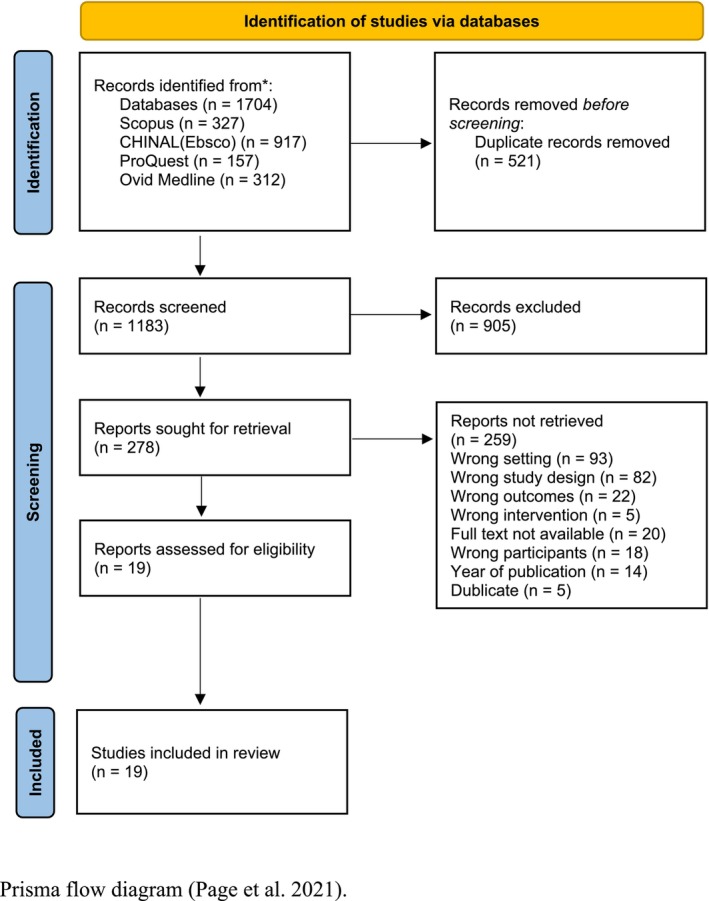
Prisma flow diagram.

Five researchers (HO, HK, EJ, KM, and JJ) participated in the screening process. A total of 1704 publications were retrieved from the database searches; after 521 duplicate publications were removed, the total number of studies was 1183. The next step was to screen original studies by titles and abstracts, based on which 905 studies were eliminated. Next, a full text screening of 278 studies was conducted, and 259 papers did not meet the inclusion criteria and were eliminated. Each study underwent a double screening process, and conflicts were resolved by a third reviewer. A total of 19 articles met the inclusion criteria and were subjected to quality appraisal. The Prisma Flow diagram presents the screening process (Figure 1).

### 3.4 Quality Appraisal

For quality appraisal assessment, the JBI critical appraisal tool was used for analytical cross‐sectional studies (Moola et al. 2020) to assess the methodological quality of each study (*n* = 19). Three studies scored eight points, nine scored 6–7 points, and two scored five points. It is important to note that five studies scored only 3–4 points out of eight (see Supporting Information S2). Overall, the methodological quality of the studies was mostly good. To ensure rigour, three researchers (HO, KM, and JJ) were involved in the quality appraisal assessment. Each study underwent a double assessment separately, and disagreements were discussed and resolved through consensus. The quality assessment did not influence whether the original study would be included in the review.

### 3.5 Data Extraction and Analysis

Data extraction was carried out before a synthesis was created, and the data was tabulated (Table 2). All included studies were extracted by authors, year of publication, country of origin, study purpose, participants, methodology (design, instrument, and measuring scale, data analysis), exposure of interest (factors associated with outcome), outcomes, and key results (Munn et al. 2022). The key results were based on the data from the included studies and the research question of this review. A narrative synthesis was employed to analyse the data, which combines the findings of the original studies selected for the systematic literature review (Popay et al. 2006) to form a coherent narrative synthesis of the quantitative studies (Campbell et al. 2018).

**TABLE 2:** Data extraction.

Authors et al., year, country	Study purpose	Participants	Methodology: design, instrument, scale (e.g., Likert scale 1–4), data analysis	Exposure of interest: factors associating with outcome	Outcomes	Key results
Baumann et al. 2018, Canada	The aim of this study was to analyse nurses' perceptions of the impact of an extended transition programme on key dimensions of care delivery 1–6 years after graduation	Nurse graduates (*n* = 2369) who graduated between 2007 and 2012 and registered on the employment portal	Cross‐sectional study. Convenience sampling method. Instruments: Data were obtained from the Policy Impact on Nurse Employment and Practice web‐based survey, which included demographic and employment information. The survey was pre‐tested. Sixteen survey items were selected as outcome variables and clustered into five key dimensions of care delivery. New graduate nurses were asked to rate how helpful their orientation was for each item using a 5‐point Likert scale. The data were analysed using multiple linear regression analysis. Descriptive statistics are reported as mean and standard deviation (SD) for continuous variables and as proportions for categorical variables	Extended transition programme	New graduate nurses' commitment to the organisation and profession	Results indicated that respondents who participated in the transition programme had statistically significantly higher mean scores on level of confidence, level of comfort, clinical decision‐making, providing safe patient care and performance/competence compared to respondents who did not participate in the transition programme. Respondents who participated in the transition programme had statistically significantly higher mean scores on communication with other nurses, patients and families, physicians and the health care team than those who did not. Results of the regression analysis for care management demonstrated that respondents who participated in the transition programme scored significantly higher on prioritising patient care and time management. Respondents who participated in the programme rated their commitment to the organisation and to the profession significantly higher. Early graduates rated commitment to the profession higher than recent graduates did, while commitment to the organisation was marginally significant for the year of graduation
Boamah and Laschinger 2015, Canada	The purpose of this study was to test a hypothesised model linking perceptions of workplace empowerment and psychological capital (PsyCap) to new graduate nurses' work engagement by integrating theories of empowerment, PsyCap and work engagement	Newly graduated nurses (*n* = 205) with less than 2 years experience in acute care hospitals	Longitudinal study. A hypothesised model was tested using data from the second wave of a longitudinal study of newly graduated nurses. Instruments: Conditions of Work Effectiveness‐II (CWEQ‐II), a 5‐point Likert scale; PsyCap was measured using the subscales of the Psychological Capital Questionnaire (PCQ), a 6‐point Likert scale; The short version of the Utrecht Work Engagement Scale (UWES), a seven‐point Likert scale. The data were analysed with descriptive statistics, and inferential statistical analyses for the major study variables demographics were conducted. The hypothesised model was tested using multiple linear regression analysis	The combination of personal and organisational resources (PsyCap and workplace empowerment)	New graduate nurses' work engagement	The hypothesised model was supported. The combined effect of workplace empowerment and PsyCap explained 38% of the variance in new nurses' work engagement. Workplace empowerment and PsyCap were significant independent predictors of work engagement
Choi and Yu 2022, South Korea	This study aimed to examine novice nurses' perception of the effects of preceptors' mentoring function on the former's self‐efficacy and organisational commitment.	Novice nurses (*n* = 160) from Korean general hospitals who had worked for less than a year after completing their preceptorship	Cross‐sectional study design. Convenience sampling method. Instruments: The mentoring function measurement tool, a 5‐point Likert scale; A self‐efficacy measurement tool, a 5‐point Likert scale; Organisational commitment tool, a 5‐point Likert scale. The data were analysed using post‐test, ANOVA, descriptive statistics, independent t‐tests, Pearson's correlation coefficient, and multiple regression analysis. Data normality was obtained by skewness and kurtosis for the multivariate test	Novice nurses' mentoring (Preceptors' mentoring function), preceptorship training period, novice nurses' work experience, marital status	Novice nurses self‐efficacy and organisational commitment	The preceptors' mentoring function as perceived by the novice nurses was 3.87, the self‐efficacy of the novice nurses was 3.71 points, and the organisational commitment was 3.46 out of 5 points. The results of the multiple regression analysis showed that mentoring function significantly affected novice nurses' self‐efficacy (*β* 0.50, *p* < 0.01) and organisational commitment (*β* = 0.54, *p* < 0.01). Further, the preceptorship training period significantly affected organisational commitment (*β* = 0.13, *p* < 0.05)
Cottle‐Quinn et al. 2022, Australia	The aim of this work is to identify factors that impact early career nurses' intentions to remain in their current position and compare them with what impacts intentions to remain in the profession	Final‐year nursing students (Phase One *n* = 293, The final sample retained from Phase Three *n* = 194.) enrolled in undergraduate nursing programmes in Australia from two universities	Prospective cohort survey design. Convenience sampling method. Instruments: Early Career Nurse Employment Experience Survey, which included adaptations of the Casey‐Fink Readiness for Practice Survey and Casey‐Fink Nurse Retention Survey; the Work Environment, Support and Encouragement scale from the Casey‐Fink Nurse Retention Survey; the original Casey‐Fink Nurse Retention Survey, a 5‐point Likert scale; Researcher‐designed questions. The data were analysed using Cronbach's alpha coefficient, binary logistic and multiple regression, and the Pearson product–moment correlation coefficient. Responses to open‐ended questions were coded, classified, and counted, with a higher number suggesting a more prominent category	Work Environment, Support and Encouragement and Stress in Personal Life	Nurses' intentions to remain	The professional turnover rate was 6.7% in total. Higher work environment, support and encouragement scores, and stress in personal life were the only predictors of my intention to remain in the profession. Statistical modelling could not predict the intention to remain in the current position
Failla et al. 2021, USA	The purpose of this article is to describe how nurse leaders in 1 large healthcare system designed, implemented, and evaluated a system‐wide program to enhance the NGNR experience, ensure clinical competence, and facilitate organisational enculturation and commitment. The 2nd purpose is to discuss the findings that a 1‐year NRP exposes nurse residents to vulnerability in leaving the organisation	Newly Graduated Registered nurses (baseline, *n* = 117; 12 months, *n* = 100; and 24 months, *n* = 97) across 5 hospitals	Descriptive, comparative study design. Instruments: Allen and Meyer Affective Commitment, a 5‐point Likert scale; Casey‐Fink Graduate Nurses Experience, a 4‐point Likert scale; Eisenberger Social Support, a 4‐point Likert scale; Grey‐Toft and Anderson Nursing Stress Scale, a 5‐point Likert scale; Halfer‐Graf Job/Work Environment Nurse Satisfaction, a 4‐point Likert scale; Intent to Leave, a 5‐point Likert scale; Organisational Citizenship Behaviour Norms, a 5‐point Likert scale; Pearson Civility Norms, a 5‐point Likert scale; Pisanti Self‐efficacy, a 5‐point Likert scale; Spector Preceptor Evaluation (Primary Preceptor), a 5‐point Likert scale; Spector Preceptor Evaluation (Secondary Preceptor), a 5‐point Likert scale. The data were analysed using descriptive statistics and variance analysis. Nonparametric data were analysed using frequencies, percentages, and *χ* ^2^	Social support, stress, civility, and job satisfaction	New graduate nurses' affective commitment and intention to leave	Findings demonstrated a reduction in affective commitment and increased nursing stress from baseline to 12 and 24 months of employment. The highest mean in intent to leave occurred at 12 months, highlighting the vulnerability of the NGNR at that time
Fernet et al. 2017, Canada	To examine whether different forms of motivation (the reasons for which nurses engage in their work) predict intention to quit the occupation and organisation through distinct forms (affective and continuance) and targets (occupation and organisation) of commitment	French Canadian newly registered nurses (*n* = 572) working in public health care	Cross‐sectional study design. Instruments: The hypothesised model was tested by structural equation modelling, The Revised Motivation at Work Scale, and a 7‐point Likert scale. Occupational and organisational turnover intentions were assessed using a single item each: I am thinking about leaving my current healthcare facility (organisational turnover intention), and I am thinking about leaving the nursing profession (occupational turnover intention). Each item was scored on a 7‐point Likert scale. The data were analysed using hierarchical regression analyses and root mean square error of approximation (RMSEA), and standardised root mean square residual (SRMR)	Autonomous and controlled motivation	New graduate nurses' affective commitment and intention to leave	Autonomous motivation (nurses accomplish their work primarily out of pleasure and satisfaction or because they endorse the importance or value of their work) negatively predicts intention to quit the profession and organisation through target‐specific affective commitment. However, although controlled motivation (nurses accomplish their work mainly because of internal or external pressure) is positively associated with continuance commitment to the occupation and organisation, it directly predicts, positively so, intention to quit the occupation and organisation
Heidari et al. 2017, Iran	The objective of this study is to find personal factors (physical, mental–emotional, social) and organisational factors (job stress, social support, and job satisfaction and organisational factors) that influence nursing staff retention	New graduate nurses (*n* = 500)	Autonomous motivation (nurses accomplish their work primarily out of pleasure and satisfaction or because they endorse the importance or value of their work) negatively predicts the intention to quit the profession and organisation through target‐specific affective commitment. However, although controlled motivation (nurses accomplish their work mainly because of internal or external pressure) is positively associated with continuance commitment to the occupation and organisation, it directly predicts, positively so, intention to quit the occupation and organisation	Job stress, social support, and job satisfaction and organisational satisfactions	Nurse retention	The first multilevel regression analysis, based on the 18 diaries with 580 entries, showed that complexity of care, lack of support and lack of competence were negatively related to novice nurses' affective commitment, whereas received support was positively related. The next multilevel regression analyses showed that all contextual, relational and cognitive factors were related to negative or positive emotions
Hoeve et al. 2018, The Netherlands	The aim of the study was to investigate whether contextual, relational and cognitive factors derived from novice nurses' work experiences are related to positive and negative emotions and affective commitment	New graduate nurses (*n* = 18) with bachelor's degree in nursing, aged under 30 and with no more than 1 year's work experience	A multilevel repeated measures within‐subjects design. Convenience sampling method. Instrument: Nurses' diaries and data were collected from weekly measurements. The diary entries (*n* = 580) and the data were inductively explored using content analysis. The texts were deductively coded. The codes were quantified, and frequencies were calculated. The nurses completed a short survey; affective commitment to the profession was measured with three items derived from the Repeated Exploration and Commitment Scale, a 6‐point Likert scale. Multilevel analysis was conducted. The data were analysed using three multilevel regression analyses	Complexity of care, support from colleagues, supervisors and physicians and perceived competence, lack of support, lack of competence	Novice nurses' emotions and affective commitment	The first multilevel regression analysis, based on the 18 diaries with 580 entries, showed that complexity of care, lack of support and lack of competence were negatively related to novice nurses' affective commitment, whereas received support was positively related. The next multilevel regression analyses showed that all contextual, relational and cognitive factors were either related to negative or positive emotions
Kenny et al. 2016, Australia	This paper investigates the links between satisfaction with nursing education and job satisfaction, and job dissatisfaction and intentions to leave a nursing job	Registered nurses (*n* = 204) were employed in their first job after graduating from the Bachelor of Nursing (BN) program at one of two Australian universities	A longitudinal cohort study design. Instruments: The survey was developed specifically for the study. “What do you expect to be doing 1 year from now?” Response options: (1) Working as a nurse with the current employer; (2) Working as a nurse with a different employer; (3) Working in a field other than nursing; (4) Not working. Satisfaction with nursing education was measured with six questions a 5‐point Likert‐type response. Job satisfaction was measured with nine questions, a 5‐point Likert‐type response, except satisfaction with work hours, which had response options “Yes” or “No.” The current analysis uses data from the first survey completed after graduation. The data were analysed using structural equation modelling (SEM) and generalised structural equation modelling (GSEM)	Work environment satisfaction and work hours and wages satisfaction, Participating in a graduate transition program	Job satisfaction in nursing	Two job satisfaction sub‐scales were identified: (1) work environment satisfaction and (2) work hours and wages satisfaction. Work preparation satisfaction was significantly and positively associated with both job satisfaction scales, but only work environment satisfaction was significantly associated with the expectation to stay in the job; a one standard deviation increase in work environment satisfaction was associated with a 13.5 percentage point reduction in the probability of expecting to leave. The estimated effect of satisfaction with education on expecting to leave, occurring indirectly through job satisfaction, was small (reducing the probability by less than three percentage points for a 1‐point increase in work preparation satisfaction). Participating in a graduate transition program had the most significant effect, reducing the average probability of expecting to leave by 26 percentage points
Kim and Choi 2022, South Korea	This study aimed to identify the effects of preceptors' teaching behaviour, resilience, and organisational socialisation on the intention of new graduate nurses to stay	New graduate nurses (*n* = 167) worked at a university hospital for 3 months to 1 year	Quantitative descriptive study design. Instruments: The clinical teaching behaviour inventory (CTBI), a 5‐point Likert scale; The resilience of new graduate nurses tool, a 5‐point Likert scale; The organisational socialisation of new graduate nurses tool, a 5‐point Likert scale; Intention to stay tool, an 8‐point Likert scale. The data were analysed using descriptive statistics, independent t‐test, one‐way ANOVA, Pearson correlation coefficients and a double mediating effect analysis	Preceptors' teaching behaviour, resilience, organisational socialisation	New graduate nurses' intention to stay	No significant differences were noted in the effects of all general characteristics on the intention to stay of participants. Preceptors' teaching behaviour positively correlated significantly with resilience, organisational socialisation, and intention to stay. In addition, resilience showed significant positive correlations with organisational socialisation and intention to stay. Organisational socialisation also showed a significant positive correlation to stay. Preceptors' teaching behaviour had direct positive effects on the resilience of new graduate nurses, and resilience had direct positive effects on organisational socialisation and intention to stay. Additionally, the organisational socialisation of new graduate nurses significantly affected their intention to stay. However, the direct effects of preceptors' teaching behaviour on the organisational socialisation of new graduate nurses were not significant. The total effect of resilience and organisational socialisation in the relationship between preceptors' teaching behaviour and intention to stay of new graduate nurses was greater than the direct effects of preceptors' teaching behaviour on the intention to stay of new graduate nurses, thereby confirming the mediating effects
Koskinen et al. 2023	This study aimed to examine the association between nursing education‐related factors and NGNs' job satisfaction	The study population consisted of NGNs from ten European countries after 1 year of work experience (*n* = 2792)	Cross‐sectional study design, convenience sampling method. Instruments: Job satisfaction was measured with three questions: satisfaction with current job, quality of care in the workplace, and nursing profession using a 4‐point Likert scale. In addition to demographic information (age, gender, country), nursing education‐related factors (data at graduation point, T1) were used as background factors: (1) satisfaction with the nursing education program (very unsatisfied–very satisfied), (2) level of study achievements (very poor–excellent), (3) nursing as the 1st study choice (yes/no), (4) intention to stay in nursing (never–very often), and (5) generic nursing competence (very low–very high) evaluated with the Nurse Competence. Scale (NCS). The NCS was used as a background factor, dividing the NGNs into three groups on the basis of their total Visual Analogue Scale (VAS) score at graduation (rather good: VAS mean < 50, good: VAS > 50–75, and very good: VAS > 75–100). Continuous and normally distributed data were summarised using mean and standard deviation (SD) and categorical variables with counts and percentages. The data were analysed using logistic regression analysis	Nursing education related factors	Newly graduated nurses' job satisfaction	Finnish, German, Lithuanian and Spanish NGNs' satisfaction with the nursing education program at graduation was statistically significantly associated with their job satisfaction, i.e., satisfaction with their current job, the quality of care, and the nursing profession. Moreover, NGNs who had fairly often or very often intention to stay in nursing at graduation were more satisfied with their current job, with the quality of care, and with the nursing profession compared with NGNs who had never or fairly seldom intention to stay in nursing at graduation
Moss 2022, USA	This study's purpose was to determine the impact of participation in the NANNP formal mentoring program on job satisfaction and retention for novice and experienced NNPs in an academic medical center in Tennessee	Participants included new graduate APPs and experienced NNPs (*n* = 40)	Cohort study design, convenience sampling method. Instruments: The electronic survey consisted of 3 demographic questions regarding nursing experience (both RN and NP), as well as years of employment in current position; the 44‐item MNPJSS, a 6‐point Likert scale, and 2 intent‐to‐stay items, a 6‐point Likert scale. Data were analysed using a 1‐way repeated‐measures analysis of variance and a Spearman rank order correlation	Participation in a mentoring program	Newly graduated nurses' job satisfaction and intention to stay	Project results identified a significant difference in MNPJSS scores for participants in a 6‐month formal mentoring program using the NANNP tool kit. The MNPJSS scores moderately correlated with intent to stay at 1 year and strongly correlated with intent to stay at 3 years
Nagai et al. 2022, Japan	This study aims to examine the causal relationship between the personal and professional resources for nurses to work vigorously (PPR‐N) and work engagement among nurses in the early stages of their careers, considering time as a key mediating factor	Staff nurses with at least 10 months of work experience (*n* = 1204)	A longitudinal study design, snowball sampling method. Instruments: The Utrecht Work Engagement Scale, a 7‐point Likert scale; Personal and Professional Resources for Nurses to Work Vigorously (PPR‐N), a 7‐point Likert scale. Data were analysed using structural equation modelling	Personal and professional resources for nurses to work vigorously	Newly graduated nurses' work engagement	PPR‐N significantly and positively affected work engagement after 3 months among early‐career nurses with less than 10 years of nursing experience. The PPR‐N is a reliable antecedent of work engagement, which is typical of early‐career nurses
Owings and Gaskins 2020, USA	The purpose of this study was to evaluate a 12‐month nurse residency program based in a community hospital setting	Newly graduated (*n* = 121) nurses with less than 1 year of clinical experience matriculated within 6 months of employment	Quantitative, retrospective study design. Nonprobability, convenience sampling method. Instrument: Casey–Fink survey, Likert‐type responses. The data were analysed using repeated‐measures analysis of variance (ANOVA), Welch one‐way ANOVA procedures, multiple paired *t* tests and Descriptive statistics (frequencies and percentages)	Communication and leadership skills, technical skills proficiency, perception of social support, ability to organise and prioritise patient care, less fearful of causing harm, more confident in the nursing role, high levels of professional satisfaction	Nurse residency program ('s effect on newly graduated nurses)	Participation in the NRP positively influenced newly graduated nurses' communication and leadership skills, ability to organise and prioritise patient care, technical nursing skills, and perceptions of social support. Participants were less fearful of causing harm, more confident in the nursing role, and reported high levels of professional satisfaction throughout the 12‐month program. Turnover rates of the study participants dropped with each cohort and were half the turnover rate of newly graduated nurses who did not participate in the NRP
Pfaff et al. 2014, Canada	The purpose of this paper is to describe the team and organisational factors that promote NG nurse engagement in collaborative practice	Nurses (*n* = 514) who had graduated from a baccalaureate nursing program and had been employed in a registered nurse position for 3 years or less	A mixed methods study design. Criterion, maximum variation and snowball sampling strategies. Phase one: an exploratory cross‐sectional survey of NG nurses. The second phase is a qualitative study and a semi‐structured interview. Instruments: a demographic questionnaire; Collaborative Practice Assessment Tool (CPAT), a 7‐point scale. Data were analysed using descriptive statistics (frequencies, means and standard deviations), student t‐tests, Pearson correlation and analyses of variance, and multiple linear regression. Qualitative data were transcribed verbatim and coded. The codes were clustered and reduced through categorical aggregation	Organisational and team facilitators. (Team and Interprofessional Respect, support and opportunities, Organisational resources for Leadership and Quality preceptor and mentor programs)	Newly graduated nurses' engagement	The team and organisational predictors of NG engagement in collaborative practice were as follows: satisfaction with the team (*p* < 0.000), number of team strategies (*p* < 0.000), participation in a mentorship or preceptorship experience (*p* < 0.000), accessibility of manager (*p* < 0.001), and accessibility and proximity of educator or professional practice leader (*p* < 0.001 and *p* < 0.002, respectively). Qualitative analysis revealed the team facilitators to be respectful, team support and face‐to‐face interprofessional interactions. Organisational facilitators included supportive leadership, participation in a preceptorship or mentorship experience and time
Sugawara et al. 2023, Japan	This study examined the relationship between self‐compassion and turnover intention among early career nurses in Japan, as well as the mediating effects of nursing job stress, burnout, and work engagement. This study also examined whether low stress predicts work‐engagement and high work‐engagement predicts low turnover intention	Nurses who had worked for 5 years or less (*n* = 326)	Cross‐sectional study design. Instruments: Nursing Job Stressor Scale (NJSS), a 4‐point Likert scale; Japanese Burnout Scale (JBS), a 5‐point Likert scale; a 3‐item version of the Utrecht Work Engagement Scale, a 7‐point Likert scale; the Turnover Intention Scale, a 4‐point Likert scale. Data were analysed using structural equation modelling	Self‐compassion, nursing job stress, burnout and work engagement	Newly graduate nurses' turnover intentions and work engagement	Self‐compassion was negatively associated with nursing job stress and burnout and positively associated with work engagement. Moreover, conflict with other nursing staff and quantitative work was positively associated with burnout, whereas qualitative work was negatively associated with work engagement. However, nursing role conflict was negatively associated with burnout and positively associated with work engagement. Burnout predicted turnover intention, whereas work engagement did not
Tarhan et al. 2021, Turkey	This study aimed to determine the relationship between the attitudes of nurse‐physician collaboration and the levels of intention to leave the current job and professional commitment among NGNs	Full‐time nurses, having at least a bachelor's degree in nursing, and having professional experience less than 3 years (*n* = 274)	Cross‐sectional study design. Instruments: a personal information form, the Jefferson Scale of Attitudes towards Physician‐Nurse Collaboration, a 4 point Likert scale; Intention to Leave Scale, a 5 point Likert scale and Nursing Professional Commitment Scale, a 4 point Likert scale. Data was analysed using Kolmogorov–Smirnov test and Spearman's rank correlation coefficient	Nurse‐physician collaboration	Intention to leave the current job and professional commitment among NGNs	NGNs had a high positive attitude towards nurse‐physician collaboration with a median score of 49 (45–54). The professional commitment of NGNs was high level with a median score of 75 (69–86). The intention to leave the current job median score was 3 (2.3–3.6) out of 5. There was a statistically significant correlation between attitudes towards nurse‐physician collaboration and the levels of intention to leave the current job (*r*s = −0.22; *p* < 0.01) and professional commitment (*r*s = 0.42; *p* < 0.01)
Walker and Campbell 2013, Australia	The purpose of this study is to investigate the relationship between work readiness and a number of variables that capture the work experiences of graduate nurses during their first year of practice: job satisfaction, work engagement, and intention to remain. The present study also explores whether job satisfaction and engagement mediate the relationship between work readiness and intention to remain	Graduate nurses (*n* = 96) across two regional hospitals in Victoria, recruited during graduate nurse study days at their place of work, graduate programme length was 1 year	Cross‐sectional study design. Instruments: revised Work readiness scale (WRS), a 10‐point Likert scale; Job satisfaction scale, a 5‐point Likert scale; a shortened version of the Utrecht Work Engagement Scale (UWES), a 7‐point Likert scale; Intention to remain scale, a 7‐point Likert scale. Data were analysed using multiple regression analyses, The Baron and Kenny (1986) method for testing mediation, the Sobel test and Bootstrapping. The means, standard deviations, inter‐correlations and reliability coefficients for all variables were tested in the analyses	Organisational acumen, clinical competence and social intelligence	Graduate nurses' work engagement	Three of the four work readiness dimensions (organisational acumen, clinical competence and social intelligence) were found to predict job satisfaction and work engagement. Moreover, both job satisfaction and work engagement were found to mediate the relationship between organisational acumen and intention to remain. The findings indicate that dimensions of work readiness uniquely predict work outcomes
Yu et al. 2021, Taiwan	The purpose of this study is to identify the factors influencing the intention to stay of newly graduated male nurses. This was explored using a structural equation model (SEM). Based on the Job Demands‐Resources (JD‐R) model, this study tested the model that social support, resilience, and nursing professional commitment influence the intention to stay and the mediating effect of nursing professional commitment in the above relationship	Newly graduated male nurses (*n* = 272) who have a clinical seniority of 2 years or less after graduating	Cross‐sectional study design. Purposive and snowball sampling methods. Instruments: Personal Resource Questionnaire (PRQ2000), a seven‐point Likert‐type scale (where 1 indicated ‘strongly disagree’ and 7 ‘strongly agree’); Connor‐Davidson Resilience Scale (CD‐RISC), a five‐point Likert‐type scale where 0 indicated ‘not true at all’ and 4 ‘true all the time’; Professional Commitment Scale, a five‐point Likert‐type scale (where 1 indicated ‘strongly disagree’ and 5 ‘strongly agree’); Intention to Stay in Nursing questionnaire, a 10‐point Likert scale. Data were analysed using the SEM methodology to test the proposed model using the model fit criteria. In addition, CFA was used to evaluate the factor structures for the instrument	Professional commitment, social support, resilience	Newly graduated male nurses' intention to stay	The hypothetical model had a good fit with the data. Nursing professional commitment had a complete mediating effect between social support and intention to stay and between resilience and intention to stay. The nursing professional commitment was highly positively correlated to the intention to stay

## 4 Results

### 4.1 Study Characteristics

Factors affecting the engagement of newly qualified nurses in the workplace were explored in 19 original studies (see Table 2). The original studies selected for the systematic review were published between 2013 and 2023 and conducted in Australia (3), Canada (4), Iran (1), Japan (2), The Netherlands (1), Taiwan (1), Turkey (1), the United States of America (3), and one international study (1). The designs of the selected studies were cross‐sectional (*n* = 10), cohort (*n* = 3), mixed methods (*n* = 2), longitudinal (*n* = 2), descriptive comparative study design (*n* = 1), and quantitative retrospective study design (*n* = 1). All studies collected data with survey questionnaires. Participants in the original studies were newly graduated nurses who had been working for less than 3 years. The number of participants varied from 18 to 2792 (total *n* = 10,244).

### 4.2 Factors Affecting the Engagement of Newly Qualified Nurses

In the included studies, newly qualified nurses' work engagement was assessed using various measures, including newly graduated nurses' commitment to the organisation and profession (Baumann et al. 2018; Failla et al. 2021; Fernet et al. 2017; Choi and Yu 2022; Hoeve et al. 2018; Tarhan et al. 2021), newly graduated nurses' work engagement (Boamah and Laschinger 2015; Nagai et al. 2022; Pfaff et al. 2014; Sugawara et al. 2023; Walker and Campbell 2013), or newly graduated nurses' intention to stay in the profession and organisation (Cottle‐Quinn et al. 2022; Heidari et al. 2017; Kenny et al. 2016; Kim and Choi 2022; Koskinen et al. 2023; Moss 2022; Owings and Gaskins 2020; Yu et al. 2021). These findings have been categorised and tabulated according to the seven categories that explain factors associated with the engagement of newly qualified nurses: (1) supportive workplace; (2) transition and orientation to the workplace; (3) competence and career development in nursing practice; (4) personal and psychological characteristics; (5) work environment characteristics; (6) stress and challenges in the work environment; and (7) satisfaction with work. The factors associated with the outcomes are shown in Table 3.

**TABLE 3:** Factors associated with newly graduated nurses' work engagement.

	Newly graduated nurses' commitment to organisation and profession						Newly graduate nurses' work engagement					Newly graduated nurses' intentions to stay in the profession and organisation							
	Baumann et al. (2018)	Failla et al. (2021)	Fernet et al. (2017)	Choi and Yu (2022)	Hoeve et al. (2018)	Tarhan et al. (2021)	Boamah and Laschinger (2015)	Nagai et al. (2022)	Pfaff et al. (2014)	Sugawara et al. (2023)	Walker and Campbell (2013)	Cottle‐Quinn et al. (2022)	Heidari et al. (2017)	Kenny et al. (2016)	Kim and Choi (2022)	Koskinen et al. (2023)	Moss (2022)	Owings and Gaskins (2020)	Yu et al. (2021)
Participants (*n*)	*n* = 2369	Baseline, *n* = 117; 12 months, *n* = 100; and 24 months, *n* = 97	*n* = 572	*n* = 160	*n* = 18	*n* = 274	*n* = 205	*n* = 1204	*n* = 514	*n* = 326	*n* = 96	*n* = 194	*n* = 500	*n* = 204	*n* = 167	*n* = 2792	*n* = 40	*n* = 121	*n* = 272
*Factors*																			
Supportive workplace																			
Social support Support from colleagues, supervisors, and physicians Work environment, support, and encouragement Organisational socialisation Accessibility of nurse manager Number of team strategies Civility		*p* = 0.001 *p* = 0.001			*p* ≤ 0.05	*p* < 0.01 *p* < 0.01	*p* < 0.01		*p* = 0.001 *p* = 0.001			*p* = 0.017 *p* = 0.676 *p* = 0.050	no *p*‐values		*p* < 0.001			*p* = 0.028	*p* < 0.050
Transition and orientation to workplace																			
Accessibility of nurse educator Proximity of nurse educator Mentorship or preceptorship Training and orientation period Preceptor's work experience Preceptor's Position Extended transition program effect on commitment to organisation: Transition Nurse group (differences in educational preparation) Transition × nurse group (an interaction term of extended orientation and nurse group) Year of graduation Extended transition program effect on commitment to profession: Transition Nurse group (differences in educational preparation) Transition × nurse group (an interaction term of extended orientation and nurse group) Year of graduation Preceptors' teaching behaviour Satisfaction With preceptors Number of preceptors Student practice institution	*p* < 0.001 *p* = 0.40 *p* = 0.105 *p* = 0.051 *p* < 0.001 *p* = 0.26 *p* = 0.005 *p* = 0.039	*p* = 0.052		*p* < 0.001 *p* = 0.02 *p* = 0.71 *p* = 0.15					*p* = 0.001 *p* = 0.002 *p* = 0.001						*p* = 0.792 *p* < 0.001 *p* = 0.512 *p* = 0.759				
Competence and career development and in nursing practice																			
Education level Shared education Prior nursing qualification Graduate program Grade point average Undergraduate employment Access to opportunities to learn and grow Access to information for professional growth Overall work readiness and readiness for practice Social intelligence Organisational acumen Technical Skills Communication/Leadership (skills) Less fearful of causing harm Organising patient care Work experience in health care Job tenure Positive or negative patient experience Personal work characteristics Nursing practice role Job status Clinical competence Lack of competence Nursing professional commitment Accountability and responsibility of nurses			NS NS NS	*p* = 0.77 *p* < 0.01	NS *p* ≤ 0.05	*p* < 0.01 *p* < 0.01 *p* < 0.01	*p* < 0.01 *p* < 0.01	*p* < 0.001			*p* < 0.05 *p* < 0.001 *p* < 0.001 NS NS	*p* = 0.07 *p* = 0.416 *p* = 0.104 *p* = 0.659 *p* = 0.425 *p* = 0.473 *p* = 0.203		*p* = 0.017 *p* < 0.001 *p* = 0.094	*p* = 0.856 *p* = 0.379		NS	no *p*‐ value *p* = 0.000 *p* = 0.000 *p* = 0.003	*p* < 0.001
Personal and psychological characteristics																			
Autonomous and controlled motivation Total empowerment Resources Vigour Dedication Absorption Total psychological capital Hope Optimism Self‐efficacy Resilience Self‐compassion Gender Age Marital status Has children Religions Speaks English			*p* ≤ 0.05 NS	*p* < 0.001 *p* = 0.94 *p* = 0.24 *p* = 0.04 *p* = 0.10			*p* < 0.01 *p* < 0.01 *p* < 0.01 *p* < 0.01 *p* < 0.01 *p* < 0.01 *p* < 0.01 *p* < 0.01 *p* < 0.01 *p* < 0.01	*p* < 0.001		*p* < 0.01	NS	*p* = 0.239 *p* = 0.070		*p* = 0.890 *p* = 0.223 *p* = 0.007 *p* = 0.630 *p* = 0.425	*p* < 0.001 *p* = 0.428 *p* = 0.361				*p* < 0.001
Work environment characteristics																			
Work unit Work environment Desired unit Public hospital Metropolitan area Organisational factors Hours and wages				*p* = 0.10									no *p*‐values	*p* = 0.003 *p* = 0.002 *p* = 0.005 *p* = 0.831	*p* = 0.147 *p* = 0.214				
Stress and challenges in work environment																			
Work related stress Stress in personal life Occupational and organisational turnover intention Complexity of care Existential events Lack of support Conflict with other nursing staff, physicians, and patients Nursing role conflict Burnout		*p* = 0.000	*p* ≤ 0.05		*p* < 0.05 NS *p* < 0.05					*p* < 0.05 *p* < 0.001 NS *p* < 0.01 NS *p* < 0.05, *p* < 0.001 *p* < 0.01, *p* < 0.001		*p* = 0.621 *p* = 0.026	no *p*‐values					NS	
Satisfaction with work																			
Professional Satisfaction Work preparation satisfaction Job satisfaction Satisfaction with professional team Satisfaction with current job Satisfaction with the quality of care in the workplace		*p* = 0.000							*p* = 0.001		*p* < 0.01	*p* = 0.287 *p* = 0.653	no *p*‐values	*p* = 0.019		*p* < 0.0001 *p* < 0.0001 *p* = 0.0003	*p* < 0.01	NS	

#### 4.2.1 Supportive Workplace

Organisational socialisation is explained as a process of learning the work ethic, performance capacity, expected behaviours, and knowledge established within the organisation that are necessary for organisational members (Kim and Choi 2022), and social support (Yu et al. 2021) showed a significant positive correlation (*p* < 0.001) with professional commitment and intention to stay. Social support's positive influence on professional commitment (Yu et al. 2021) reduced stress and intent to leave among newly graduated nurses (Failla et al. 2021). Social support was significant (*p* = 0.001) and negatively correlated with newly graduated nurses' intentions to leave (Failla et al. 2021). Those who scored higher on the work environment support and encouragement scales predicted intention to remain in the nursing profession (in phase one, when the participants were completing their nursing degree: *p* = 0.017; in phase two, 6 months later: *p* = 0.676, and in phase three, 12 months later from the start: *p* = 0.050) (Cottle‐Quinn et al. 2022).

Support from preceptors was significant (*p* ≤ 0.05). It positively related to newly graduated nurses' commitment (Hoeve et al. 2018), and there was a significant correlation between support from preceptors, peers, and physicians with work engagement (*p* < 0.01) (Boamah and Laschinger 2015). Professional commitment had a moderate, positive, and statistically significant correlation with nurse‐physician collaboration (*p* < 0.01) and physician‐nurse relationship (*p* < 0.01) (Tarhan et al. 2021). Enjoyment working with a preceptor, head nurse support in personal problems, supervisor's support when creating a dispute with the doctor, enjoying working with colleagues, and appreciating matron during performing tasks were often effective on retention, and these factors were also positively associated with intent to stay (Heidari et al. 2017). Accessibility of manager (*p* = 0.001) and number of team strategies (*p* = 0.001) predicted significantly newly graduated nurses' work engagement in collaborative practice (Pfaff et al. 2014). Participation in a nurse residency program significantly influenced newly graduated nurses' perception of social support (*p* = 0.28) (Owings and Gaskins 2020). Also, civility had a significant (*p* = 0.001) association with newly graduated nurses' affective commitment (Failla et al. 2021).

#### 4.2.2 Transition and Orientation to Workplace

Newly graduated nurses who participated in a transition programme rated their commitment to the organisation (*p* < 0.001) and to the profession (*p* < 0.001) significantly higher compared to those who did not participate. Early graduates rated commitment to the profession higher than recent graduates did, while commitment to the organisation was marginally significant for the year of graduation (*p* = 0.051). There were statistically significant differences in commitment to organisation and profession when adjusting for the year of graduation (*p* = 0.039) and nurse group (*p* = 0.005). Length of time since graduation was added to the regression model to account for variation due to the length of time employed (Baumann et al. 2018). In addition, participation in a mentorship or preceptorship experience (*p* < 0.001), accessibility of nurse educator (*p* < 0.001), and proximity of nurse educator (*p* < 0.002) predicted significantly newly graduated nurses' engagement in collaborative practice (Pfaff et al. 2014). Organisational commitment significantly differed according to nurses' preceptorship training period (*p* = 0.02). The preceptors' mentoring function, as perceived by the participants, was significantly correlated with novice nurses' organisational commitment (*p* < 0.001). Organisational commitment was significantly affected by the preceptors' mentoring functions (*p* < 0.01) and the preceptorship training period (*p* < 0.05). (Choi and Yu 2022.) Preceptors' teaching behaviour also had significant (*p* < 0.001) and positive correlations with intention to stay (Kim and Choi 2022).

#### 4.2.3 Competence and Career Development in Nursing Practice

Newly graduated nurses' professional commitment to nursing (*p* < 0.001) influenced positively their intentions to stay (Yu et al. 2021). Overall work readiness (*p* < 0.05) was significantly correlated with work engagement (Walker and Campbell 2013), and lack of competence (*p* ≤ 0.05) was negatively related to commitment (Hoeve et al. 2018). Professional commitment also showed a positive and statistically significant, but weak, correlation with nursing role in patient care (*p* < 0.01) and with accountability and responsibility of nurses (*p* < 0.01) (Tarhan et al. 2021). Newly graduated nurses' access to opportunities to learn and grow and access to information for professional growth (*p* < 0.01) (Boamah and Laschinger 2015), social intelligence (*p* < 0.001) and organisational acumen (*p* < 0.001) (Walker and Campbell 2013) were all significantly correlated with work engagement. Also, professional commitment had a weak but positive and statistically significant correlation with shared education (*p* < 0.01) (Tarhan et al. 2021). According to Kenny et al. (2016), having a prior nursing qualification predicted a higher probability of expecting to change jobs (*p* = 0.017). Organisational commitment significantly differed according to newly graduated nurses' work experience (*p* < 0.01); the longer the work experience, the higher the organisational commitment. The highest commitment was reported by those with a work experience of 3–6 months (Choi and Yu 2022). Nurses with a minimum of 10 years of experience had significantly higher total work engagement scores than nurses with less than 10 years of experience (*p* < 0.001) (Nagai et al. 2022). Participating in a program for new graduate nurses (*p* < 0.001) was significantly associated with a reduced probability of expecting to leave the job, and being in a graduate program (*p* < 0.001) had the greatest total effect, reducing the expectation of changing jobs by 26.3%, on average. (Kenny et al. 2016). Newly graduated nurses who participated in a nurse residency programme (NRP) were more confident and comfortable performing technical skills by the end of the NRP. Newly graduate nurses' scores on the communication and leadership factor increased significantly for all three measurement periods. Participants became more confident and comfortable delegating tasks (*p* = 0.001), suggesting changes to the plan of care (*p* = 0.001), completing job responsibilities (*p* = 0.001), and caring for a dying patient (*p* = 0.001). Participants became more confident and comfortable with their ability to organise, prioritise, and complete patient care on time. By the programme's end, participants were less overwhelmed and less fearful of causing harm (*p* = 0.001) and had significantly less difficulty organising patient care (*p* = 0.003) (Owings and Gaskins 2020).

#### 4.2.4 Personal and Psychological Characteristics

Overall, workplace empowerment was significantly related to work engagement (*p* < 0.01) and psychological capital (*p* < 0.01), and these were significantly correlated with each other (*p* < 0.01). Workplace empowerment and psychological capital combined explained a significant variance in new nurses' perception of work engagement (*p* < 0.05). Both empowerment and psychological capital were significant independent predictors of work engagement (*p* < 0.01). Resources, vigour, dedication, absorption, hope, optimism, self‐efficacy, and resilience were all significant independent predictors of work engagement (*p* < 0.01) (Boamah and Laschinger 2015). Choi and Yu (2022) also noted in their study that self‐efficacy and organisational commitment were significantly correlated (*p* < 0.001). Self‐compassion had a significant positive, but weak, correlation with work engagement (*p* < 0.01), and also, the direct effect of self‐compassion on work engagement was significant (*p* < 0.01). (Sugawara et al. 2023.) Personal and professional resources were positively correlated with work engagement (*p* < 0.001) (Nagai et al. 2022). Resilience showed significant positive correlations (*p* < 0.001) and direct association (*p* = 0.008) with newly graduated nurses' intention to stay (Kim and Choi 2022). In addition to that, resilience also had a direct significant effect and a positive influence on nursing professional commitment (*p* < 0.001) (Yu et al. 2021). Different from the Kim and Choi (2022) study, resilience did not have a significant direct effect on the intention to stay (*p* < 0.05). However, nursing professional commitment significantly mediated the relationship between resilience and intention to stay (Yu et al. 2021). Motivation, autonomous and controlled, predicted significantly and positively different forms of occupational and organisational commitment (*p* ≤ 0.05) (Fernet et al. 2017). Factors regarding personal characteristics and marital status (*p* = 0.04, *p* = 0.07) were the only ones to have a significant association with newly graduated nurses' commitment to the organisation (Choi and Yu 2022) and intentions to stay in the profession and organisation (Kenny et al. 2016).

#### 4.2.5 Work Environment Characteristics

Satisfaction with the work environment was significantly associated with the expectation to leave the job; a one standard deviation increase in satisfaction with the work environment was associated with a 13.5percentage point reduction in the probability of expecting to change jobs (*p* = 0.003). Intention to leave was associated with dissatisfaction with the work environment (including staffing, support, professional development, responsibility, quality of care, and physical environment). Working in a public hospital (*p* = 0.002) and a metropolitan area (*p* = 0.005) was significantly associated with a reduced probability of expecting to leave the job (Kenny et al. 2016). Responding to fluctuations in staff workload and needs (with a significance of 98.9%) was mentioned as one of the most effective ways to influence staff retention. Organisational factors showed that factors such as salary, reward, and benefits, timely payment of salary and reward, workload, hours of work, shifts set, stress, and feedback from the supervisor had an association with staff retention (Heidari et al. 2017).

#### 4.2.6 Stress and Challenges in the Work Environment

Stress was related to newly graduated nurses' commitment to the organisation (Failla et al. 2021) and on the other hand, turnover intentions had a significant and negative correlation with work engagement (*p* < 0.01) (Sugawara et al. 2023). The decline in affective commitment was significantly and negatively related (*p* = 0.001) to the nurses' stress levels. Stress from workload had the highest mean for stress, followed by stress from conflict with physicians and stress from the death and dying of patients (Failla et al. 2021). Job stress factors such as lack of choice in selecting a favourite ward, insufficient staff, and working as an on‐call nurse in another ward were factors associated with nurse retention. Burnout showed a significant negative correlation (*p* < 0.01) and association with work engagement (*p* < 0.001) (Sugawara et al. 2023). Conflict with nursing managers, conflict with other coworkers, being criticised by nursing managers, and the death of patients also affected retention and were negatively associated with staying in the position (Heidari et al. 2017). Qualitative stress was negatively associated with work engagement (*p* < 0.001), and it correlated negatively and significantly with nurses' work engagement (*p* < 0.05). Qualitative stress included being assigned unfamiliar tasks, being asked to perform tasks outside one's capability, and not fully understanding the operation of medical equipment (Sugawara et al. 2023). The complexity of care was significantly and negatively related to newly graduated nurses' commitment (*p* < 0.05). Lack of support from colleagues, supervisors and physicians was negatively related to commitment (*p* < 0.05) and positively related to negative emotions. Negative factors (i.e., care complexity, lack of support, and perceived lack of competence) were more related to the level of commitment than the positive factors (Hoeve et al. 2018). In the Cottle‐Quinn et al. (2022) study, having stress in an early career nurse's personal life was predictive of intention to remain in the profession. The most common causes of this stress were personal relationships and finances. For those respondents who reported having stress in their personal life that impacted their work, most of the respondents noted that it hurt their work. Higher work environment, support, and encouragement scores remained predictive of wanting to remain in the profession; however, the strongest predictor was stress. Those with stress in personal life were three times more likely to remain in the profession than those without (Cottle‐Quinn et al. 2022). Also, nursing role conflict was positively correlated (*p* < 0.05) and associated with work engagement (*p* < 0.001) (Sugawara et al. 2023).

#### 4.2.7 Satisfaction With Work

Job satisfaction and organisational satisfaction influence the retention of newly graduated nurses (Heidari et al. 2017). Job satisfaction was related to the newly graduated nurses' affective commitment to the organisation (Failla et al. 2021) and the association between job satisfaction and intention to remain was significant (*p* < 0.01) (Walker and Campbell 2013). A statistically significant, strong positive correlation was noted between job satisfaction and intention to stay at 1 year and 3 years in the current position (*p* < 0.01) among newly graduated nurses who participated in a mentoring program (Moss 2022). Satisfaction with the team predicted significant engagement of newly graduated nurses in collaborative practice (*p* = 0.001) (Pfaff et al. 2014). Work preparation satisfaction was significantly and positively associated with job satisfaction scales. An increase in work preparation satisfaction was associated with an increase in work environment satisfaction (*p* = 0.001) and an increase in hours and wages satisfaction (*p* = 0.023) (Kenny et al. 2016). Newly graduated nurses who had fairly often or very often intention to stay in nursing at graduation were more satisfied with their current job (*p* < 0.001), with the quality of care in the workplace (*p* = 0.0003), and with the nursing profession (*p* < 0.001) compared with newly graduated nurses who had never or fairly seldom intention to stay in nursing at graduation (Koskinen et al. 2023).

## 5 Discussion

This systematic review identified seven categories of factors associated with newly graduated nurses' work engagement. The engagement of newly qualified nurses as an outcome was focused on newly graduated nurses' commitment to the organisation and profession, their work engagement, and their intentions to stay in the profession and organisation. The findings of this study confirm previous research, which noted that support from supervisors had an important influence on the intention to leave among nurses and support from supervisors showed significant relationships with the intention to leave (Chami‐Malaeb 2022). Providing support will enable nurses to build effective relationships with supervisors and colleagues, handle time and priority management, reduce stress and anxiety, increase self‐confidence, and develop the ability to absorb workplace cultural norms (Baharum et al. 2023). Social support can affect transition shock, suggesting that newly graduated nurses with more support from family, friends, colleagues, and supervisors reported less transition shock (Cao et al. 2021). In this study, it was found that when newly graduated nurses receive more support, their level of commitment increases and when social support goes down, intent to leave increases. This demonstrates the importance of social support to ensure newly graduated nurses' work.

Participation in transition and mentorship programmes, as well as in preceptorship programmes, increased newly graduated nurses' work engagement. Nurses in mentoring programmes are more likely to stay in the profession, adhere to professional standards, and feel positively about their work (Rudin and Ludin 2018). Preceptorship programmes also reduce turnover among newly graduated nurses and improve their relationships with preceptors (Van Zyl et al. 2021). Newly graduated nurses should participate in a nurse residency programme, and it should be the goal of every practice setting because nurse turnover can create instability in the work environment (Cadmus et al. 2023). Newly graduated nurses' transition to practice needs nursing education to teach students a realistic understanding of the transition process, address the theory–practice gap, and collaborate with hospitals. Likewise, hospitals must have realistic expectations from newly graduated nurses and distribute work according to their competencies. Hospitals must also allow newly graduated nurses sufficient time for role integration (Gautam et al. 2023).

Previous studies have found that orientation plays a critical role in newly graduated nurses' transition to the workplace (Lindfors et al. 2018; Gautam et al. 2023). To succeed in newly graduated nurses' orientation to nursing work, organisations must invest in communal commitment to the orientation processes, and organisations need to have robust professional orientation know‐how and nursing management to have skills in supportive leadership (Lindfors et al. 2018). To foster positive transition experiences, a supportive work culture is needed. This also promotes equity, respect, and safety among nurses in the workplace (Gautam et al. 2023). Successful orientation supports the transition to practice for newly graduated nurses, and supportive elements in orientation ensure possibilities to focus on good orientation practices for preceptors (Lindfors et al. 2018). Healthcare organisations need a culture of feedback, individualised orientation, and understanding that orientation should be a common interest. Investing in orientation is investing for the future (Lindfors et al. 2022a).

In this study, competence and career development were a group of factors which had conflicting results. This review provides new insights into how competencies, work readiness, or career development do not necessarily translate into a stronger commitment to nursing work. However, previous research has found that it facilitates newly graduated nurses' transition to work (Lindfors et al. 2022b). For example, work experience was a factor which had a significant effect on organisational commitment, but healthcare experience did not predict new graduate nurses' intentions to stay in the profession. Also, overall work readiness was significantly correlated with work engagement but readiness for practice did not predict newly graduated nurses' intentions to stay in the profession or in their current position.

In terms of personal and psychological factors, it was found that there were various significant independent predictors of work engagement. Those predictors were, for example, workplace empowerment, psychological capital, resources, vigour, dedication, absorption, hope, optimism, self‐efficacy, and resilience. Previous studies support this result by noting that psychological capital also correlated positively with newly graduated nurses' engagement and retention intention and lower burnout levels (Flinkman et al. 2023). A study by Xie et al. (2021) found that gender was a significant predictor of burnout among newly graduated nurses, with women experiencing higher levels of emotional exhaustion and depersonalisation compared to men, while men reported higher levels of personal accomplishment. Similarly, Park et al. (2022) observed that newly graduated female nurses were more likely to struggle with adapting to new work environments, which was associated with a higher intent to leave the profession compared to their male counterparts.

Newly graduated nurses' satisfaction with the work environment was significantly associated with the expectation to leave the job, and it reduced the probability of expecting to change jobs. Intention to leave was associated with dissatisfaction with the work environment. Li et al. (2020) support the result that the intention to leave was associated with dissatisfaction with the work environment. Dissatisfaction with the workplace was associated with newly graduated nurses' turnover intentions (Li et al. 2020). Nurses who had worked for 13–18 months, who had rotating shifts, who had orientation lasting for 13 weeks or longer, and whose hospital and department were not desired had higher levels of turnover intention compared with those who worked for less than 6 months, who had a permanent shift, and whose hospital and department were desired (Li et al. 2020). Nurses working in specialty areas showed lower intention to leave (Li et al. 2020). Newly graduated nurses with difficulty in adapting to new work environments were working at large hospitals with rotating shifts (Park et al. 2022). Nurses switching hospitals or moving to a nonhospital job were associated with the type of hospital (Kim and Kim 2022).

In this study, job stress influenced retention and was related to newly graduated nurses' commitment to the organisation. Previous studies show that occupational stress predicts newly graduated nurses' intention to leave; the more significant the occupational stress and the worse the professional identity, the more likely a newly graduated nurse has an intention to leave (Zhang et al. 2017). Stress from taking care of patients, from roles and workload, from co‐workers and daily life, and from lack of professional knowledge and skills significantly impacted turnover intention among nurses (Zhou et al. 2022). A previous study also found that issues with the work environment were related to newly graduated nurses leaving their positions (Bae 2023). Job stress is previously associated with newly graduated nurses' turnover intention, increasing the likelihood of it. Newly graduated nurses with turnover intentions had higher job stress with job demands, interpersonal conflict, and lack of reward (An et al. 2022). Previous research also shows the motivations for nurses to leave the profession: a challenging work environment, emotional distress, disappointment in nursing reality, and a culture of hierarchy and discrimination (Bahlman‐van Ooijen et al. 2023). Previous studies have examined the effects of job stress on work engagement and turnover. However, an interesting finding in this review was that stress in one's personal life predicted wanting to remain in a profession. It is possible that stress in their personal lives can make newly graduated nurses feel more comfortable at work while escaping the challenges of their personal lives. This is an exciting finding and should be explored in the future to see how personal life stress affects the work of newly qualified nurses. The link between personal life stress and work engagement brings a new approach, as the association between work‐related stress and work engagement has previously been studied.

One study differed from these results, stating that job satisfaction did not predict newly graduated nurses' intention to remain in their current positions or the profession. A previous study from Laschinger et al. (2016) stated that new nurses' job satisfaction and career retention are related to perceptions of work environment factors that support their professional practice behaviours and high‐quality patient care. Psychological capital, structural empowerment, and perceived staffing adequacy can predict job satisfaction and reduce nurse burnout (Şenol Çelik et al. 2024).

### 5.1 Limitations and Strengths

This systematic review has a limitation concerning publication bias as it only included published articles in English or Finnish. Additionally, the literature search did not include a search for grey literature. The Prisma 2020 checklist (Supporting Information S3) has been completed and implemented during this systematic review process (Page et al. 2021) to strengthen the reporting of the review. Most of the original studies have been conducted in North America and Asia, so there may be cultural differences about the other continents of nursing work. Differences in study designs, populations, outcomes, and the definition of ‘work engagement’ in the included studies can limit the ability to combine data or draw definitive conclusions. This review was conducted following the JBI guidelines for evidence synthesis, explicitly focusing on systematic reviews to ensure transparency in reporting the review process and findings. The JBI critical appraisal tool for analytical cross‐sectional studies was utilised to assess the methodological quality of the included original studies. Notably, five original studies did not reach a good methodological quality (50% or less of the total score), which can influence this study's reliability.

## 6 Conclusions

This systematic review aimed to explore the factors associated with newly graduated nurses' work engagement. Identifying and understanding these factors provides valuable insights into how to manage the attractiveness of the healthcare sector. Further research is needed to comprehensively understand nurse leaders' tools to improve nurses' work engagement in different healthcare settings. This research could be valuable to units when they want to develop and improve work engagement. To support newly graduated nurses' work engagement, nurse leaders should focus on new nurses' effective support systems in the workplace and their ability to develop and educate themselves to enhance their knowledge and skills in nursing. Additionally, the transition of newly graduated nurses from student to professional and their integration into the organisation should be considered. Organisations should have policies and procedures to ensure quality orientation, and units must implement transition and mentorship programmes. This can provide a more stable and engaged workforce for the healthcare sector. Newly graduated nurses need ways to influence work stress by reducing it. Nurse leaders need to provide a supportive working environment where new nurses feel valued and respected to improve job satisfaction. Understanding how personal and psychological factors affect nurses' work engagement to retain nurses in the profession is also essential. Individual needs and differences should be taken into account in order to retain nurses in the profession. Investing in these ways to improve work engagement can help attract and retain the nursing workforce and foster new nurses' adaptation and integration into the healthcare organisation. Reflecting on feedback from newly graduated nurses allows problems and issues to be addressed, and listening to them can benefit healthcare organisations and patients.

## Author Contributions

All authors have agreed on the final version and meet at least one of the following criteria (recommended by the ICMJE*): (1) substantial contributions to conception and design, acquisition of data, or analysis and interpretation of data; (2) drafting the article or revisiting it critically for important intellectual content.

## Conflicts of Interest

The authors declare no conflicts of interest.

## Supporting information


Data S1.

Data S2.

Data S3.

## Data Availability

The data that support the findings of this study are available on request from the corresponding author.
