# Supplementary material for: Factors Associated With Newly Graduated Nurses' Work Engagement: Systematic Review of Quantitative Studies
Source: J Adv Nurs. 2025 Jun 27;82(3):1947–72. doi: 10.1111/jan.17069 (PMC12907590; doi:10.1111/jan.17069)
Supplement: Supplementary file 1 — Data S1. [file JAN-82-1947-s001.docx]

Supplementary file 1. Search strategy used in the electronic databases

|  | **Search strategy** |
| --- | --- |
| CINAHL | ( ( ( (MH "Practical Nurses") OR nurs* ) AND ( ("new* qualified" or "new* graduate*" or newcomer* or "early career*" or novice* or "recent graduate*") ) ) OR ( (MH "Novice Nurses") OR (MH "New Graduate Nurses") ) )  AND  ( ( (MH "Work Engagement") or (MH "Personnel Turnover") or (MH "Personnel Retention") ) OR ((work OR job OR organi?ation* OR employee*) N2 (engage* OR commit*)) OR ( intent* N2 (stay* OR leav*) ) OR turnover )  NOT  ( (MH "Nonexperimental Studies+") or “qualitative research” or “observational stud*” or “observational research”) or ((MH "Intervention Trials") OR (MH "Experimental Studies+") OR (MH "Randomized Controlled Trials+") OR (“interven* or “randomi?ed controlled trial*” or experimental or “trial stud*”) OR ((MH "Qualitative Studies+") OR (MH "Systematic Review") OR (MH "Meta Analysis") or (“qualitative stud*” or “review” or “meta-analysis”) ) |
| Scopus | nurs* AND ("new* qualified" or "new* graduate*" or newcomer* or "early career*" or novice* or "recent graduate*")  AND  "Personnel Retention" OR ((work OR job OR organi?ation* OR employee*) W/2 (engage* OR commit*)) OR ( intent* W/2 (stay* OR leav*)) OR turnover  NOT  "qualitative research" or "observational stud*" or "observational research" or interven* or "randomi?ed controlled trial*" or experimental or "trial stud*" or "qualitative stud*" or review or "meta-analysis" or nonexperimental |
| ProQuest | nurs* AND ("new* qualified" or "new* graduate*" or newcomer* or "early career*" or novice* or "recent graduate*")  AND  "Personnel Retention" OR ((work OR job OR organi?ation* OR employee*) N/2 (engage* OR commit*)) OR ( intent* N/2 (stay* OR leav*)) OR turnover  NOT  "qualitative research" or "observational stud*" or "observational research" or interven* or "randomi?ed controlled trial*" or experimental or "trial stud*" or "qualitative stud*" or review or "meta-analysis" or nonexperimental |
| Ovid Medline | ((exp Licensed Practical Nurses/ or nurs*.ab,kf,kw,ti.) and ("new* qualified" or "new* graduate*" or newcomer* or "early career*" or novice* or "recent graduate*").ab,kf,kw,ti. and (exp Work Engagement/ or exp Personnel Turnover/ or (personnel retention or ((work OR job OR organi?ation* OR employee*) adj2 (engage* OR commit*)) or (intent* adj2 (stay* or leav*)) or turnover).ab,kf,kw,ti.)) not ("qualitative research" or "observational stud*" or "observational research" or interven* or "randomi?ed controlled trial*" or experimental or "trial stud*" or "qualitative stud*" or review or "meta-analysis" or nonexperimental).ab,kf,kw,ti. |
