# Supplementary material for: Factors Associated With Newly Graduated Nurses' Work Engagement: Systematic Review of Quantitative Studies
Source: J Adv Nurs. 2025 Jun 27;82(3):1947–72. doi: 10.1111/jan.17069 (PMC12907590; doi:10.1111/jan.17069)
Supplement: Supplementary file 2 — Data S2. [file JAN-82-1947-s002.docx]

Supplementary File 2. Quality assessment of the included studies using the JBI Critical Appraisal Checklist for Analytical Cross Sectional Studies

| Included studies | 1. Were the criteria for inclusion in the sample clearly defined? | 2. Were the study subjects and the setting described in detail? | 3. Was the exposure measured in a valid and reliable way? | 4. Were objective, standard criteria used for measurement of the condition? | 5. Were confounding factors identified? | 6. Were strategies to deal with confounding factors stated? | 7. Were the outcomes measured in a valid and reliable way? | 8. Was appropriate statistical analysis used? | Total points |
| --- | --- | --- | --- | --- | --- | --- | --- | --- | --- |
| Baumann et al., 2018, Canada | Yes | Yes | Yes | Yes | Yes | Yes | No | Yes | 7/8 |
| Boamah S & Laschinger H, 2015, Canada | No | Yes | Yes | No | Yes | No | No | Yes | 4/8 |
| Choi E & Yu S (2022) South Korea | Yes | Yes | Yes | Yes | Yes | Yes | Yes | Yes | 8/8 |
| Cottle-Quinn et al., 2022, Australia | No | Yes | Yes | Yes | Yes | Unclear | No | Yes | 5/8 |
| Failla et al., 2021, USA | No | Yes | Yes | No | No | No | No | Yes | 3/8 |
| Fernet et al., 2017, Canada | No | Yes | Yes | Yes | Yes | Yes | Yes | Yes | 7/8 |
| Heidari et al., 2017, Iran | Yes | Yes | Yes | No | No | No | No | Unclear | 3/8 |
| Hoeve et al., 2018, The Netherlands | Yes | Yes | Yes | Yes | Yes | Yes | Yes | Yes | 8/8 |
| Kenny et al., 2016, Australia | Yes | Yes | Yes | Yes | Yes | Yes | Yes | Yes | 8/8 |
| Kim E & Choi E, 2022, South Korea | Yes | Yes | Yes | Yes | Unclear | Unclear | Yes | Yes | 6/8 |
| Koskinen et al, 2023 | No | Yes | Yes | Yes | Yes | Yes | Yes | Yes | 7/8 |
| Moss C.R., 2022, USA | No | Yes | Yes | Yes | Yes | No | Yes | Yes | 6/8 |
| Nagai et al., 2023, Japan | Yes | Yes | Yes | Yes | No | No | Yes | Yes | 6/8 |
| Owings, C. & Gaskins, S., 2020, USA | No | Yes | Yes | Yes | Unclear | No | Yes | Yes | 5/8 |
| Pfaff et al., 2014, Canada | No | Yes | Yes | Yes | Yes | Yes | Yes | Yes | 7/8 |
| Sugawara et al., 2023, Japan | No | Yes | Yes | No | No | No | Yes | Yes | 4/8 |
| Tarhan et al., 2022, Turkey | Yes | Yes | Yes | Yes | No | No | Yes | Yes | 6/8 |
| Walker A. & Campbell K., 2013, Australia | No | Yes | Yes | No | No | No | Yes | Yes | 4/8 |
| Yu et al., 2021, Taiwan | Yes | Yes | Yes | Yes | Yes | No | Yes | Yes | 7/8 |
